# Prooxidant Mechanisms in Iron Overload Cardiomyopathy

**DOI:** 10.1155/2013/740573

**Published:** 2013-11-19

**Authors:** Ching-Feng Cheng, Wei-Shiung Lian

**Affiliations:** ^1^Department of Medical Research, Tzu Chi General Hospital and Department of Pediatrics, Tzu Chi University, Hualien, Taiwan; ^2^Institute of Biomedical Sciences, Academia Sinica, Taipei, Taiwan

## Abstract

Iron overload cardiomyopathy (IOC), defined as the presence of systolic or diastolic cardiac dysfunction secondary to increased deposition of iron, is emerging as an important cause of heart failure due to the increased incidence of this disorder seen in thalassemic patients and in patients of primary hemochromatosis. At present, although palliative treatment by regular iron chelation was recommended; whereas IOC is still the major cause for mortality in patient with chronic heart failure induced by iron-overloading. Because iron is a prooxidant and the associated mechanism seen in iron-overload heart is still unclear; therefore, we intend to delineate the multiple signaling pathways involved in IOC. These pathways may include organelles such as calcium channels, mitochondria; paracrine effects from both macrophages and fibroblast, and novel mediators such as thromboxane A2 and adiponectin; with increased oxidative stress and inflammation found commonly in these signaling pathways. With further understanding on these complex and inter-related molecular mechanisms, we can propose potential therapeutic strategies to ameliorate the cardiac toxicity induced by iron-overloading.

## 1. An Introduction to Iron Overload Cardiomyopathy 

Heart failure due to iron overload can develop either as a result from repeated blood transfusions seen in thalassemias or hereditary hemochromatosis. The most important clinical conditions leading to iron overload cardiomyopathy (IOC) is thalassemia major, in which heart failure remains the major cause of death (60%), greatly exceeding deaths from infection (13%) and liver disease (6%) [1]. In patients of thalassemias and hemoglobinopathies, aggressive transfusion therapy and iron chelation have been palliatively applied to them before the 80's in order to improved their quality of life and decreased the morbidity and mortality rates associated with these diseases [2]. The only curative treatment for these diseases, bone marrow transplantation (BMT) was first successfully used to treat a thalassemic patient in 1982 [3]. Since then, more than 1000 patients have been treated with BMT around the world [4] and the outcome of these transplants from centers in Europe, North America, and Asia was around 80% of patients who survive long term and of these nearly 90% are cured of their diseases [5]. The risks of BMT using HLA-identical sibling donor could be predicted in advance according to the presence or absence of only three criteria: hepatomegaly, evidence of portal fibrosis, and inadequate iron chelation with existence of hemochromatosis. The latter could result in IOC and hepatic damages. Even for thalassemic patients, who have successfully undergone BMT and acquired normal hematologic status, the most important long-term consequences for those patients would be the complications associated with their moderate-severe iron overload [6].

IOC, defined as the presence of systolic or diastolic cardiac dysfunction secondary to increased deposition of iron, is emerging as an important cause of heart failure due to increased incidence of this disorder seen in thalassemic patients and in patients of primary hemochromatosis [7, 8]. Clinical iron-overload leads to a classical cardiomyopathy of restrictive physiology with severe diastolic dysfunction, but preserved systolic function in the early stages of the disorder that correspond with lower concentration of iron were reported by researchers in National Taiwan University Hospital [9]. Moreover, in the later stages with high levels of iron, heart failure progress quickly with severe systolic dysfunction, and ventricular dilation in these patients. Death ensues promptly in the later stages due to heart failure and/or severe arrhythmia [7, 10]. Although the exact mechanism of iron-induced heart failure remains to be elucidated, excess iron-catalyzed free radical generation and cytotoxic aldehyde production are believed to play a role in damaging the myocardium and altering cardiac function [11]. Due to the complication of chronic transfusion and ineffectiveness to reverse the course of iron overloading even in post-BMT patients of thalassemia major and/or hereditary hemochromatosis, iron overload heart diseases are commonly seen among these patients. At present, only palliative treatment by regular desferrioxamine administration was recommended to these patients whereas its effect is setback by both high cost and poor patient compliance in the long term period; or its limited therapeutic effect on severe iron-overload condition.

During iron overload, transferrin, the carrier of iron in the circulation, which is normally 30% saturation, becomes fully saturated, and the toxic nontransferrin bound iron species appear in the circulation [12]. Uptakes of the latter in hepatocytes, cardiac myocytes, and endocrine gland cells lead to tissue iron overload [13]. The pathophysiology of IOC is clearly mediated by reactive oxidative stress whereby the cytoplasmic iron pool become available for fenton-type reactions, leading to the conversion of Fe^2+^ into Fe^3+^ generates free radicals including the highly reactive hydroxyl radicals, in which leading to increased peroxidation and damage into lipids, proteins, and nucleic acids, triggering cellular damage and depletion of antioxidants [14]. Although the production of hydroxyl radical and lipid peroxidation are important in initiation of IOC [15], it is likely that no single signaling pathway can account for its complex pathophysiology.

## 2. Iron Enters Cardiomyocytes through Calcium Channel in IOC

In iron overload conditions, nontransferrin-bound iron (NTBI) enters the cardiomyocytes through L-type Ca^2+^ channels (LTCC) and divalent metal transporter and leads to iron-overload cardiomyopathy [16–18]. The Fe^2+^ induced slowing of Ca^2+^ current inactivation results in a increase in the time integral of the Ca^2+^ current and the net Ca^2+^ influx, which may possibly contribute to the impaired diastolic function observed during the early stages of iron overload [19, 20]. With higher concentration of Fe^2+^ associated with diseased progression, Ca^2+^ influx became decreased as competing with ferrous ion, contributing to systolic dysfunction that is characteristic of more advanced IOC. Cardiac excitation-contraction coupling are highly sensitive to changes in cellular redox state leading to reduced systolic and elevated diastolic function characteristic of IOC. Using a patch clamp technique, it has been shown that the iron current competes with the calcium current, and is inhibited by a calcium channel blocker [17, 21]. Other in vivo studies, using chronic iron overload mice models, demonstrated the inhibitory effect of LTCC blockers on cardiac iron uptake, showing decreased tissue iron content [14, 18], preserve cardiac function, and improved survival. Recent studies from Kumfu et al., showed that the use of efonidipine, a proposed specific T type Ca channel (TTCC) blockers, can lower mortality, prevented myocardial iron deposition and oxidative stress with improved cardiac function in their iron overloaded thalassemic murine model [22]. The work of Kumfu et al. actually finds that LTCC blockers were as effective as putative TTCC blockers, and more important, the TTCC blockers they used is nonspecific and blocks LTCC with a potency that is very similar to TTCC. Although TTCC are specifically confined to the SA and AV nodes in the normal healthy hearts, TTCC expression and currents are re-expressed in the ventricular myocytes under pathological conditions. However, the administration of verapamil, an LTCC blocker, likely resulted in negative inotropic effects and hypotension, which may explain the lack of a mortality benefits in iron-overload mice. Therefore, future studies are needed to provide more definitive evidence for a role of the TTCC in iron-overloaded cardiomyopathy. These findings from Kumfu et al. indicated that, unlike ferrous ion, ferric ion (Fe^3+^) uptake in cultured thalassemic cardiomyocytes is not mediated by LTCC, DMT1, or TTCC, suggesting that another alternative pathway could play a major role in thalassemic heart cells [23]. As LTCC is located in Ca^2+^-release channel/ryanodine receptor complexes in cardiomyocytes and ryanodine receptor (RYR2) are very sensitive to oxidation; therefore, Fe^2+^ entry via calcium channels is expected to have direct effect on RYR2 function and Ca^2+^ homeostasis [24, 25]. In addition, a recent paper by Rose et al. demonstrated that chronic iron loading could selectively reduce Ca(V)1.3-mediated LTCC leading to bradycardia, slowing of electrical conduction, and atrial fibrillation in patients with IOC [26]. 

## 3. Reactive Oxidative Stress and Mitochondria Pathway in IOC

In iron overloaded cardiac cells, free (redox active) iron that catalyzed the formation of highly toxic reactive oxidative species (ROS) can damage intracellular lipid, proteins, and DNA [27]. These iron driven oxidation events required that the metal interacts with cellular reducing and oxidizing equivalents such as superoxide and hydrogen peroxide, in which mitochondrial electron transport chain is a major source for supplying these pools of electrons. Therefore, mitochondrial dysfunction is likely to occur in IOC [28]. Recent studies from Gao et al. reported that increased iron exposure to cardiac myocyte cell lines could result in progressive loss of mitochondrial DNA, with decreased mRNA and protein activity for complexes I, III, and IV and mitochondrial respiration. Their followed-up in vivo study using iron dextran injection mice models showed similar results with 60–70% loss of mRNA encoded by mitochondrial DNA, yet with no change in mRNA abundance for nuclear-encoded respiratory subunits [29]. Cochrane et al. reported that oxidant-induced damage to naked DNA and intracellular DNA is greatly enhanced by iron [30, 31]. In the absence of transition metals such as iron and copper, DNA is quite un-reactive with oxidants such as H_2_O_2_ whereas, in the presence of iron, oxidative DNA scission occurs readily [32, 33]. Compared to nuclear DNA, mitochondrial DNA is more sensitive to oxidant damage [34, 35]. The reason may include that mitochondria can generates ROS and mtDNA lacks histones to protect DNA from oxidant damage. In addition, repair to mtDNA is less effective as compared to nuclear DNA. The authors suggested that chronic iron overload leads to cumulativeiron-mediated damage to mtDNA and impaired synthesis of mitochondrial respiratory chain subunits. The resulting respiratory dysfunction may partly explain the slow progression of IOC. 

The mechanism of iron acquisition by mitochondria of cardiac cells was further elucidated by Shvartsman et al. [36]. They used online fluorescence monitoring on iron for tracing the mobility of the metal from medium to cell cytosol and mitochondria in rat primary cardiomyocytes [37]. The results indicate that mitochondria rapidly taken up iron supplied to the cells as NTBI form and that the cytosolic iron traffic to the mitochondrial organelles could not be abolished by iron chelators. Under iron overload condition, it is apparently that mitochondria has limited ability to relieve themselves from labile iron accumulation, thus result in oxidative stress and ensuring damages to these critical organelles. 

## 4. Reactive Oxidative Stress Associated Inflammation and Fibrosis in IOC

Excess iron injures cells primarily by catalyzing the production of ROS in excess of the capacity of cellular antioxidant systems. These ROS cause lipid peroxidation, oxidation of amino acids with consequent protein-protein fragmentation, and DNA damage. Therefore, the effect of chelation therapy can remove excessive iron from body and also scavenge and tightly bind labile iron to prevent the generation of ROS [38, 39]. In our prior studies, we intend to use G-CSF to treat chronic heart failure induced by IOC in the hypothesis that G-CSF can mobilize endogenous stem cells in which has been reported to offer beneficial effect to acute myocardial infarction. Although our data showed that G-CSF can mobilize autologous stem cells in the IOC mice, on the contrary, G-CSF supplement worsened the IOC induced cardiac dysfunction through aggravating iron induced oxidative stress, and cardiac inflammatory profiles with systemic leukocytosis [40]. The cardiac pathology of the G-CSF added IOC heart demonstrated ventricular fibrosis with macrophages infiltration. In addition, immune-histochemical analysis revealed increased tissue factor expression and colocalization with macrophage markers CD13 [40].

Our results showing that G-CSF can promote inflammatory profiles in IOC that leads to cardiac dysfunction, are in contrast to previous reports showing G-CSF therapy to be beneficial in acute myocardial infarction [41–44] and chronic cardiomyopathy induced by doxorubicin toxicity [45]. One explanation for these disparate results could be that chronic iron loading increases oxidative stress [46]. Although G-CSF recruits hematogenic stem cells, a simultaneous induction of macrophage and tissue factor gathering “gears up” the pro-inflammatory state and drives the inflammation-fibrosis circuit. Similar study that G-CSF exacerbates cardiac fibrosis after rat myocardial infarction with increasing circulating WBCs, neutrophils, and monocytes were also reported [47, 48]. In addition to IOC, heart remodeling and failure is persistent even with optimal chelation therapy in some of the *β*-thalassemic patients. Such clinical observation may let us raise questions on whether thalassemic cardiac dysfunction can occur in the absence of transfusion related iron-overload and myocardial iron deposition. In vivo evidence was provided by a recent study by Stoyanova et al. [49]. They used Hbb^d3th/d3th^ gene deleted mouse, a mouse model closely reproduced human *β*-thalassemia major or intermedia disease, and echocardiography to follow their cardiac function longitudinally for 6 months without blood transfusion [50, 51]. These mice first demonstrated anemia associated compensated hypertrophy, then, developed age-dependent deterioration of left ventricular contractility and dysfunction that led toward decompensated heart failure. The histopathology revealed cardiac remodeling with increased interstitial fibrosis, but virtual absence of myocardial iron deposits. This study suggested that another paracrine and cardiomyocyte independent mechanism may be involved in cardiac fibrosis seen in the thalassemic hearts. 

## 5. Macrophage and Arachidonic Acid Associated Paracrine Pathway in IOC

Recent studies further demonstrated that iron overload could enhance arachidonic acid release and eicosanoids production in cultured cardiomyocytes, and suggested a causal connection between these signals electromechanical abnormalities in iron-overload cardiomyopathy [52]. Nevertheless, limit information is available regarding the downstream signaling alterations. Because expression of both PGI2 and TXA_2_ can be found in heart tissue, and PGI2 and its analogue have been reported to exert beneficial effect is cardiac ischemic injury [53]; therefore, we hypothesize that TXA_2_ may be the major eicosanoids that mediates the iron overload induced cardiomyopathy. 

**Figure 1:**
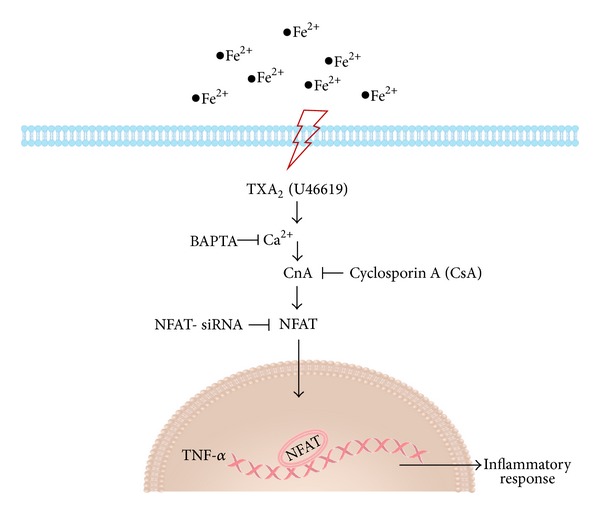
TXA_2_ mediates iron overload cardiomyopathy through TNF-*α* associated calcineurin-NFAT signaling pathway. The schematic diagram depicted that iron loading can activate TXA_2_ and induces TNF-*α* production in which can be inhibited by NFAT-SiRNA, calcineurin inhibitor (CsA), or calcium chelator (BAPTA). Addition of TXA_2_ agonist, U46619, can facilitate nuclear translocation of NFAT, thus increase proinflammatory marker TNF-*α* expression. These findings demonstrate a novel molecular mechanism of TXA_2_ in mediating iron overdosed cardiomyopathy and the involvement of calcineurin-NFAT signaling cascades in cardiac chronic inflammation.

Using iron-overload mouse model, along with TXAS gene deleted mouse, recent study from Lin et al. set out to elucidate the role of TXA_2_ in cardiac iron-overload cardiomyopathy [54]. This study first demonstrated that iron loading can induce TXAS and its product TXB2 expression in mouse heart. Second, they found that the development of iron-induced cardiac fibrosis required TXAS product TXB_2_, which is inflammation dependent. Their data showed that attenuation of iron deposition was found in the TXAS KO hearts, suggesting that TXAS is involved in the iron deposition itself in the heart. Because iron appears to be accumulated mainly in nonmyocytes macrophage cells located in the interstitial space, in which also has TXAS expression [55]. Therefore, we suggested that activated macrophages that taken up iron have increase TXAS expression, thus activated the TP receptor in cardiomyocytes in a paracrine TXA_2_-TP signaling manner. This study also demonstrates that a lack of TXAS can attenuate cardiac fibrosis and inflammation in IOC with an accompanying decrease in cellular infiltration in tissue and an attenuation of WBC, monocytes, lymphocytes, and neutrophil numbers in blood, suggesting that leukocytes-cardiomycytes TXAS-TP signaling is sufficient to induce cardiac iron deposition and activate chronic inflammation in heart. They further discovered that the TXA_2_ analogue, U46619, induces TNF-*α* production in which can be inhibited by NFAT-SiRNA, calcineurin inhibitor (CsA), or calcium chelator (BAPTA). These findings demonstrate a novel molecular mechanism of TXA_2_ in mediating iron-overdosed cardiomyopathy and the involvement of calcineurin-NFAT signaling cascades (see Figure 1). 

Because chronic iron loading may activate more than one signaling pathway that damaged the heart tissue; therefore, it is possible that iron may deposit on cardiac myocytes, and it may also stimulate macrophage infiltration and activate inflammation. In prior study by Lian et al., some of the IOC mice after G-CSF supplementation demonstrated increased ROS production with recruitment of macrophages, in which further aggravated inflammatory infiltration which eventually triggered cardiac thrombosis in the left ventricular chamber [41]. 

## 6. Novel Adiponectin Signaling Pathways Involved in IOC

Adiponectin (APN) is a circulating adipose-derived cytokine that may act as an antioxidative and anti-inflammatory protein. Although APN has been reported to confer cytoprotective effects in acute cardiac diseases, its effects in IOC are unknown. Recent studies have found a negative correlation between the levels of serum ferritin and APN [56–58], which suggests that adipocyte iron negatively regulates APN transcription via FOXO-1-mediated repression [59]. These data indicated that the increased tissue iron stores are sufficient to increase serum ferritin and decrease serum APN levels. Iron loading induced oxidative stress with the overexpression of proinflammatory molecules, such as IL-6, MCP-1, TNF-*α*, and ICAM-1, in heart or blood vasculature [60], leading to endothelial and cardiac dysfunction. Therefore, it is plausible that decreased APN levels are a risk index in cardiac inflammation and associated endothelial dysfunction.

**Figure 2:**
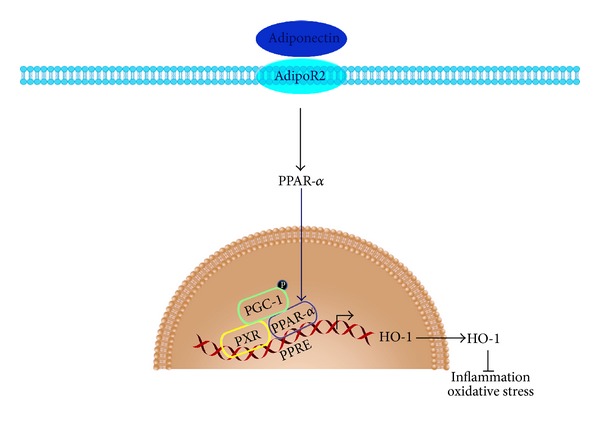
Adiponectin ameliorates cardiac inflammation in IOC through the PPAR*α*-PGC1-dependent signaling pathway. The schematic diagram showing that adiponectin exerts its beneficial effects in cardiomyocytes through the PPAR*α*-dependent HO-1 signaling pathway and requires PPAR*α*-PGC-1 interaction. The adiponectin-AdipoR2-PPAR*α* signaling may be the major pathway in exerting anti-inflammatory and antioxidative stress effects that ameliorate iron-overload cardiac dysfunction.

Our recent study aimed to investigate whether APN offers beneficial effects in iron-induced chronic heart failure [61]. IOC mice exhibited decreased left ventricular contraction and decreased serum APN levels in which the phenotype can be rescued by. in vivo cardiac AAV8-APN supplement. A further in vitro study showed that APN induced heme oxygenase-1 (HO-1) expression through the PPAR*α*-HO-1 signaling pathway. In addition, the APN-mediated beneficial effects were PPAR*α*-dependent as the APN-mediated protective effects on attenuating iron deposition were abolished in PPAR*α*-knockout mice. Lastly, we demonstrated that PPAR*α*-HO-1 signaling involved PPAR*α* and PGC-1 binding and nuclear translocation, and their levels of expression can be increased after APN therapy. These data showed that APN ameliorated iron deposition in the heart through a PPAR*α*-PGC-1-dependent mechanism and exert beneficial effects to IOC (see Figure 2).

## 7. Other Beneficial Effects against ROS and Inflammation in IOC

HMG-CoA reductase inhibitors, or statins, are known to improve cardiac dysfunction through their anti-inflammatory and antioxidative action. Statins also affect endothelial function through the production of nitric oxide [62, 63]. A recent study from Lian et al. demonstrates that simvastatin can reduce the myocardial iron depositionin G-CSF treated iron-overload heart [40]. Simvastatin administration also reduced the expression of the pro-inflammatory markers ICAM-1, tissue factor, MCP-1, and TNF-*α*. This study also revealed that simvastatin exerts their beneficial effects through elevation of both eNOS and phosphorylates Akt activity, thus ameliorates the inflammation-fibrosis found in the iron-overload heart.

A prior study by Grandel et al. showed that endotoxin depressed the contractility of isolated rat hearts by inducing TNF-*α* synthesis, and that TNF-*α*-induced microcirculatory dysfunction in mouse liver is dependent on TP receptor [64, 65]. These findings suggest that TXA_2_ may act as a paracrine facilitator of TNF-*α* expression. A recent study from Lin et al. revealed that the addition of the TXA_2_ agonist, U46619, to cardiac cultured cells can increase TNF-*α* expression [54]. This expression can be suppressed by BAPTA, CsA, and SQ29548, respectively. These results show that the TXA_2_-mediated TP receptor-calcium/calcineurin signaling pathway activates the pro-inflammatory marker TNF-*α*. Iron-overloaded mice administered the TNF-*α* antibody infliximab showed decreased TXAS expression in the heart with improved left ventricular contractility. These data imply that blockade of TNF-*α* in vivo can decrease TXA_2_ expression and attenuate IOC.

## 8. Conclusion

**Figure 3:**
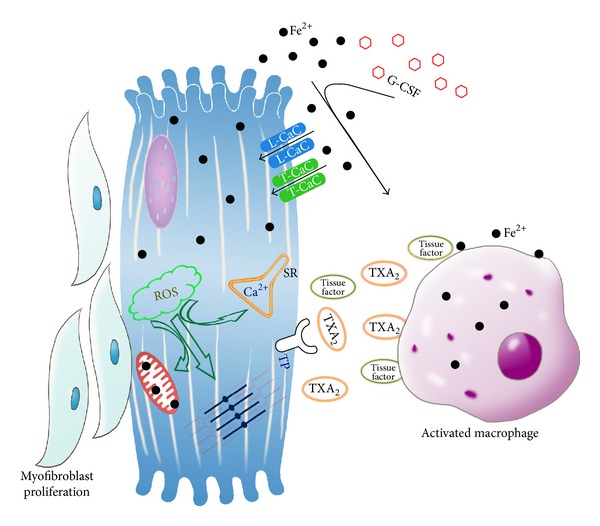
Involvement of multiple signaling pathways and cardiomyocyte-macrophage interactions in iron overload cardiomyopathy. The schematic diagram showed that iron can enter the cardiomyocytes through both L-type and T-type calcium channels and increased the ROS within cardiac cells. These effects then inhibit the calcium influx, impaired the excitation-contraction coupling and myofilaments contractility, and damage the intracellular organelles, including mitochondria. In addition, iron can activate the TXA_2_-TP receptor signaling pathway and promote cardiomyocyte-macrophage interaction. With G-CSF supplement, such macrophages recruitment and tissue factor induction will be enhanced, which further increased the ROS and gear up the inflammation-fibrosis circuit, result in aggravation of IOC and cardiac fibrosis.

In this review, we delineated the multiple signaling pathways involved in IOC. These pathways may include organelles such as calcium channels, mitochondria, paracrine effects from both macrophages and fibroblast, and novel mediators such as thromboxane A2 and adiponectin. Because iron is a prooxidant, the involved signaling pathways were associated with increased oxidative stress and inflammation. A schematic diagram (Figure 3) was depicted to show these complex and interrelated molecular mechanisms: iron can enter the cardiomyocytes through both L-type and T-type calcium channels and increased the ROS within cardiac cells. These effects then inhibit the calcium influx, impaired the excitation-contraction coupling and myofilaments contractility, and damage the intracellular organelles, including mitochondria. In addition, iron can activate the TXA_2_-TP receptor signaling pathway and promote cardiomyocyte-macrophage interaction. With G-CSF supplement, such macrophages recruitment and tissue factor induction will be enhanced, which further increased the ROS and gear up the inflammation-fibrosis circuit, result in aggravation of IOC and cardiac fibrosis. With further understanding on the molecular mechanisms involved in IOC, we propose the potential in using adiponectin, statins, and possible TNF*α* blockers for future therapeutic trials to ameliorate the cardiac toxicity induced by iron-overloading.
